# Deep-Learning-Based Stress Recognition with Spatial-Temporal Facial Information

**DOI:** 10.3390/s21227498

**Published:** 2021-11-11

**Authors:** Taejae Jeon, Han Byeol Bae, Yongju Lee, Sungjun Jang, Sangyoun Lee

**Affiliations:** 1 Department of Electrical and Electronic Engineering, Yonsei University, Seoul 03722, Korea; jtj7587@yonsei.ac.kr (T.J.); paulyongju@yonsei.ac.kr (Y.L.); jeu2250@yonsei.ac.kr (S.J.); 2 Department of Artificial Intelligence Convergence, Kwangju Women’s University, 45 Yeodae-gil, Gwangsan-gu, Gwangju 62396, Korea; kwu_BHB@kwu.ac.kr

**Keywords:** deep learning, stress recognition, stress database, spatial attention, temporal attention, facial landmark

## Abstract

In recent times, as interest in stress control has increased, many studies on stress recognition have been conducted. Several studies have been based on physiological signals, but the disadvantage of this strategy is that it requires physiological-signal-acquisition devices. Another strategy employs facial-image-based stress-recognition methods, which do not require devices, but predominantly use handcrafted features. However, such features have low discriminating power. We propose a deep-learning-based stress-recognition method using facial images to address these challenges. Given that deep-learning methods require extensive data, we constructed a large-capacity image database for stress recognition. Furthermore, we used temporal attention, which assigns a high weight to frames that are highly related to stress, as well as spatial attention, which assigns a high weight to regions that are highly related to stress. By adding a network that inputs the facial landmark information closely related to stress, we supplemented the network that receives only facial images as the input. Experimental results on our newly constructed database indicated that the proposed method outperforms contemporary deep-learning-based recognition methods.

## 1. Introduction

People in contemporary society are under immense stress due to various factors [1]. As stress is a cause of various diseases and affects longevity, it is vital to keep it under control [2,3,4]. A system that detects a user’s stress level in real time and provides feedback about how to lower stress is the need of the hour [5,6,7]. To develop such a system, high-accuracy stress recognition technology is required. In response to this need, research on stress recognition technology has been actively conducted. Reliable stress recognition technology will be useful in various fields, such as driver stress monitoring [8,9] and online psychological counseling.

Most stress-recognition studies have been conducted using a two-class classification, which divides subjects into stressed or relaxed, or using three classes, i.e., low, medium, and high stress [10]. Several stress recognition studies have been conducted on physiological signals acquired through wearable devices [8,11,12,13,14,15,16,17]. Physiological-signal-based approaches effectively recognize human stress because they use signals that immediately reveal a person’s condition, such as respiration rate, heart rate, skin conductivity, and body temperature. However, this method involves additional costs because a special wearable device is required to acquire physiological signals, which users may find too expensive or feel reluctant to wear.

Other studies have identified and classified stress using life-log data such as mobile app usage records obtained from smartphones [18,19,20,21]. As smartphones are always attached to their users, it is possible to ascertain the user’s status by accumulating data over a certain period. This approach is suitable for recognizing stress over a specific period, but fails to recognize an instantaneous stress state. By contrast, images, such as thermal images showing blood flow and respiratory rate and visual images portraying body movements and pupil size, can be used for stress recognition [22,23,24]. Some stress-recognition studies use only visual images, especially facial images, which have the advantage of only requiring a camera; the subjects need not wear additional equipment [25,26]. However, in many of these methods, handcrafted features continue to be used. In some recent studies, a neural network with handcrafted features is used in the feature extraction process [27,28,29].

Some recent studies have recognized stress using only deep learning. Zhang et al. [30] proposed a deep-learning-based method that detects the presence or absence of stress using the video footage of a person watching a video clip that induces or does not induce stress. In this method, when the face-level representation was first learned, an emotion recognition network was used to learn the emotion change between the two frames with the largest emotion difference. Furthermore, the action-level representation was learned by using motion information and an attention module that passes the entire feature through one fully connected layer. The resolution of both the facial image and upper body image was 64 × 64, which rendered the detection of small facial changes difficult.

By contrast, our study focuses on a more difficult task: subdividing stressful situations into low-stress and high-stress situations. Furthermore, the attention used was subdivided into spatial and temporal attention, and since it had a precise structure, it could be advantageously used for learning attention for each purpose. Additionally, the face-level representation was learned using all frame information, and the resolution of the facial image used was 112 × 112, which was more advantageous for detecting small facial changes. Moreover, since the proposed method does not use motion information, it can show higher performance in situations where only a face is visible or for people without bodily motion. The experimental results in Section 5.4 show that the proposed method could detect overall spatial and temporal changes in the face related to stress and that it is superior to the method presented in the previous work [30].

In a previous study [31], we constructed a database and performed deep-learning-based stress recognition using facial images. In this database, data were acquired in both the speaking and nonspeaking stages. However, this resulted in a challenge: the learning proceeds in such a way that the network classifies speaking and nonspeaking states. Moreover, the amount of data was insufficient for detecting minute changes in the face because images were stored at a rate of about five images per second. Furthermore, the stress recognition network was not designed in detail to find minute changes in facial expressions, but was instead designed as a combination of a convolutional neural network (CNN) and a deep neural network (DNN) with a simple structure.

Therefore, in this study, the database construction and network design were improved so as to alleviate the aforementioned concerns. High-quality data were acquired by designing a more sophisticated scenario, and the recognition model also had a more sophisticated design. We acquired additional data because a large-capacity image database is required to use deep learning, but there is no existing database that can be used for stress recognition. Therefore, we built a large image database by conducting a stress-inducing experiment and released the database publicly. We propose a deep-learning-based stress-recognition method using facial images from this stress recognition database.

In the proposed method, we used time-related information, which is unavailable in still images. Given that our database contains images captured from video data, we use a temporal attention module that assigns a high weight to frames related to stress when viewed from the time axis. Furthermore, we used a spatial attention module that assigns a high weight to the stress-related areas in the image to improve the performance further. One study [32] found that peoples’ eye, mouth, and head movements differ when under stress. Therefore, to accurately capture these movements, a network that receives facial landmark information was added. Accordingly, we supplemented the network, which receives only facial images as the input. In addition, designing a proper loss function when using the deep-learning method is crucial. Therefore, we designed a loss function that is suitable for our database and trained the proposed method end-to-end.

Our contributions are as follows:

1. We built and released a large-capacity stress recognition image database that can be used for deep learning;
2. We applied a multi-attention structure to the deep learning network, and the proposed method was trained end-to-end;
3. We trained a feature with stronger discriminating power by adding a network that uses facial landmarks.

The remainder of this paper is organized as follows. In Section 2, previous studies related to stress recognition and deep learning are described. In Section 3, we introduce the construction process and contents of our database. In Section 4, the proposed method is presented in detail. In Section 5, the experimental settings are described and the experimental results are analyzed. Finally, Section 6 concludes this study.

## 2. Related Work

### 2.1. Facial-Action-Unit-Based Stress Recognition Methods

Many studies have attempted to recognize stress using facial action unit information that defines the movements of the eyes, nose, mouth, and head [25,32,33,34]. There are several types of facial action units, and among them, units that are highly related to stress, such as inner brow raise, nose wrinkle, and jaw drop, are used often. In previous studies, the movement of each facial action unit was used as a feature, and classical classifiers such as random forest and support vector machine (SVM) were used for classification. Some studies recognized stress primarily using pupil size [24,35]. The pupil diameter and pupil dilation acceleration were used as features, and the SVM and decision tree were used as classifiers. Pampouchidou et al. [36] recognized stress using mouth size as a primary characteristic. Stress was recognized using normalized openings per minute and the average openness intensity obtained from mouth openness. In another study, stress was recognized by observing breathing patterns through changes in the nostril area [27]. After discovering breathing patterns through temperature changes near the nostrils, two-dimensional respiration variability spectrogram sequences were constructed using these data and were used to recognize stress. Giannakakis et al. [37] recognized stress based on facial action unit information obtained from nonrigid 3D facial landmarks, the histogram of oriented gradients (HOG), and the SVM. The limitations of the aforementioned methods are that they cannot utilize the changes in the facial colors and the full facial image because the entire image information is not used.

### 2.2. Facial-Image-Based Stress Recognition Methods

In one popular method of recognizing stress using facial images, unlike the facial action unit, a comprehensive feature is extracted from the entire image. In some studies, the HOG features were extracted from the eye, nose, and mouth regions in RGB images and used as features [26,29]. In these methods, a CNN and a method combining the SVM and slant binary tree algorithm were used as classifiers. Some studies used features extracted from thermal images or nearinfrared (NIR) images [9,22,38]. In the methods using thermal images, stress was recognized based on the tissue oxygen saturation value extracted from the thermal image or by applying a CNN to the thermal image itself. In the method using NIR images, stress recognition was performed using an SVM after extracting scale-invariant feature transform (SIFT) descriptors around facial landmarks. In other studies, stress was recognized by fusing RGB and thermal images [28,39,40]. In these methods, stress was recognized using the features extracted from super-pixels and local binary patterns on the three orthogonal plane (LBP-TOP) descriptor. All the methods introduced above used handcrafted features, but there was also a method using deep learning. This method recognizes stress by fusing facial images and motion information such as hand movements [30]. In this method, optical flow images were used to obtain motion information, and stress was recognized by applying attention to facial features and motion features. Most of the facial-image-based stress recognition studies have used handcrafted features. Many image recognition studies have shown great performance improvement through deep learning. If deep learning is used, the stress recognition performance can be further improved because stress-related high-dimensional features can be learned from images. Recently, a study [30] that recognized stress using deep learning came out, and we also tried to recognize stress using deep learning for better performance.

### 2.3. Facial-Image-Based Emotion Recognition Methods

Many studies on facial-image-based emotion recognition are being conducted, and there are similarities between emotion recognition and stress recognition studies since emotion and stress are related. Among studies on emotion recognition methods, many studies using facial landmark information are underway [41,42,43]. As changes in facial expressions are highly correlated with changes in facial landmarks, these studies input the coordinates of facial landmarks directly into a network or images created from facial landmarks. Palestra et al. [44] classified emotions using a random forest classifier after extracting geometrical features from facial landmark information. Studies on recognizing emotions in videos are also being actively conducted. For such emotion recognition, various methods for using time-related information are being studied. These include a method that uses a 3D-CNN [41,45] and a method that combines a 2D-CNN and a recurrent neural network (RNN) [42,43]. Furthermore, many recent deep-learning-based studies have improved the recognition performance by using simple modules such as the attention module [46,47]. The attention module creates attention maps that are multiplied by the input feature maps and then refines those feature maps to improve recognition performance. For example, Zhu et al. [48] proposed a hybrid attention module comprising a self-attention module and a spatial attention module to detect regions with large differences in facial expressions. Meng et al. [49] proposed a frame attention module that assigns higher weights to frames with higher importance among multiple frames when video data are input. The difference between our method and the above methods is that the former were designed to detect overall spatial and temporal changes in the face. First, attention was divided into spatial attention and temporal attention to emphasize spatially and temporally important parts, respectively. We then designed a network that could effectively detect facial changes by using preprocessed facial landmark images. We showed that the proposed method is superior to other methods through various ablation studies and performance evaluation experiments.

## 3. Database Construction

Several databases [50,51] containing data for stress recognition are available, but most contain physiological signal data; few have image-related information. As far as we know, there is only one database, i.e., the SWELL-KW database [51], that includes facial image information. This database provides four types of information: computer interactions, facial expressions, body postures, and physiology. It provides four pieces of information related to facial expressions. First, the orientation of the head in three dimensions is provided. Second, ten pieces of information related to facial movements, such as gaze direction and whether the mouth is closed, are provided. Third, 19 pieces of information related to facial action units such as inner brow raise, nose wrinkle, and chin raise are provided. Finally, probability values are provided for eight emotions such as neutral, happy, and sad. However, this database does not provide images, but only the above high-level information obtained from images. Therefore, this database cannot be used for deep-learning-based stress-recognition methods that take images as the input.

**Figure 1:**
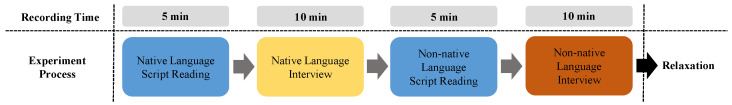
Progress of the designed stress-inducing experimental scenario, including the recording time for each stage.

Therefore, a new database is required to recognize stress using deep learning, so we built a large image database. The database we built consists of the subject’s facial images and information on whether the subject’s stress level belongs to one of three levels (neutral, low stress, or high stress). As this study involved human participants, our database was built with the approval of the Institutional Review Board of Yonsei University, and the study was conducted upon it. We created this database by designing an experimental scenario that included stress-inducing situations. The designed stress-inducing experiment scenario is depicted in Figure 1.

As research results indicated that an interview induces stress in the subject [52,53] and that the subject is stressed when asked to use a non-native language [54,55], the experimental scenario was designed in accordance with these studies’ results. Therefore, the stress-inducing situation comprised interviews in native and non-native languages. The former was established as a situation that induces low stress and the latter as a situation that induces high stress. We recruited subjects near our school. As most of the population is Korean, Koreans were selected as test subjects, and accordingly, Korean was used as the native language. English was selected as the non-native language because it is the most popular non-native language used by Koreans.

Situations in which the test subject reads scripts written in the native or non-native languages were used as the comparison group. These were considered situations that did not cause stress (i.e., neutral). If the nonspeaking situations were set as a comparison group, the network can learn to classify speaking and nonspeaking situations. Thus, the comparison group was limited to situations in which subjects read scripts. The experiment time for each stage was 5 min for the native and non-native language script reading and 10 min for the native and non-native language interviews. We set the experiment time for each script-reading stage to 5 min because we designed both script-reading stages to be stress-free so that the sum of the experiment time of the two stages would be the same (10 min) as the other stress-inducing stages in the experiment. We shot a single video at each experimental stage for each subject. As there were four experimental steps, the number of videos for each subject was four.

We collected data by recruiting 50 men and women in their 20 s and 30 s. We chose this age group because the experimental stages included reading scripts and interviewing in a non-native language. We believed that this task would be difficult for older people. In addition, the population in their 20s and 30s in the subject recruitment area was large. During the experiment involving situations that do and do not induce stress, the subject’s appearance was photographed using a Kinect v2 camera.

The data acquisition environment was as follows. The data were acquired in a windowless location so that the lighting could be kept constant. The camera was set so that only a white wall appeared behind the subject, eliminating any potential interference from a complex background. The camera was positioned in front of the subject so that the subject’s frontal face could be photographed. To ensure that the subject’s face would always be visible, hair or accessories other than glasses were not allowed to cover the subject’s face. The reason for this constraint is that if hair or accessories cover the face, they interfere with the observation of the subject’s facial changes. We enforced these constraints because the purpose of this study is to detect overall spatial and temporal changes in the face related to stress. The resolution of the recorded video is 1920 × 1080. When the data were acquired, about 24 images were saved per second, and the entire database comprises 2,020,556 images. The summary information about the database construction settings and database contents is depicted in Appendix A.

As presented in Table 1, this database comprises a large number of images for deep learning, which is considered highly useful, and was released as the Yonsei Stress Image Database on IEEE DataPort (https://dx.doi.org/10.21227/17r7-db23 (accessed on 8 November 2021)). It is publicly available for stress recognition research. We measured the stress recognition accuracy after labeling the acquired data according to the scenario we designed. We labeled the data acquired during the native language interview as low stress, the data acquired during the non-native language interview as high stress, and the data acquired while reading the script produced in the native language or non-native language as neutral.

We annotated the data in this manner because many stress recognition studies still use this method [10]. The reason why this labeling method continues to be popular is that it is difficult to annotate stress data in real time. In the case of an emotion database, an annotator can examine the facial expression of a subject and label the subject’s emotions as positive or negative in real time. This is possible because in the case of facial expressions, the emotion is visually apparent, and therefore, other people can judge to some extent whether it is positive or negative. However, in the case of stress, it is difficult to judge it solely from facial expressions. For example, while a subject may actually be stressed, it may not be evident from his/her facial expressions, or he/she may fake a smile. Therefore, many studies have created a stress-inducing situation, and all data obtained from that situation were labeled as corresponding to a stress state. We trained and tested how accurately the proposed method and other methods classified data into these three labels, and the performance of each method was compared using the test accuracy. The ablation studies and comparative experiments conducted using the established database are described in Section 5.

## 4. Proposed Methodology

In this section, we describe the structure of the proposed method for recognizing stress using facial information and multiple attention. We look at the proposed method’s overall structure and then look at the spatial attention module, facial landmark feature module, temporal attention module, and loss function, in that order.

### 4.1. Overall Structure

**Figure 2:**
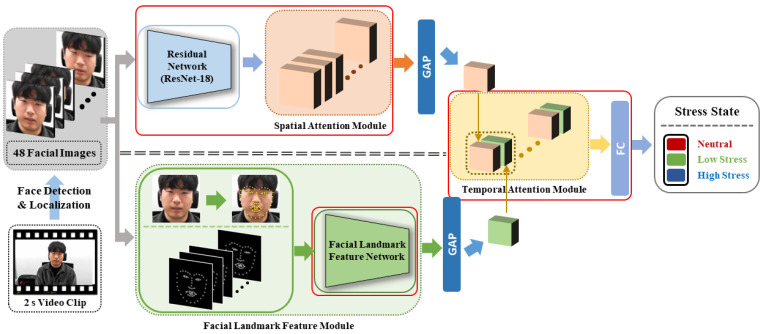
Flowchart of the proposed method. The residual network ResNet-18 extracts feature maps from facial images. GAP: global average pooling; FC: fully connected layer.

The proposed method predicts a person’s stress level from video data based on facial information. A flowchart for the proposed method is depicted in Figure 2, and the details are described below.

First, one clip was entered as the input for the proposed method. This clip was created by dividing all 5 or 10 min videos acquired in the database construction experiment into 2 s clips. As the data acquisition rate was 24 frames per second (fps), one clip consisted of 48 frames, and we used all 48 frames as the input. The size of the original image was 1920 × 1080, but when training and testing, the face area was detected, cropped, and resized to 112 × 112. A multitask cascaded convolutional network [56] was used to detect and localize the facial area. When the facial image passes through the ResNet-18 residual network [57], feature maps are generated. Furthermore, as these feature maps pass through the spatial attention module and global average pooling (GAP) [58], a facial image feature is generated.

In the spatial attention module, a high weight was assigned to the positionally important parts of the feature maps, and a lower weight was assigned to the positionally unimportant parts of the feature maps. The details of the spatial attention module are described later in Section 4.2. When a facial image passed through the facial landmark detector, 68 facial landmarks were obtained. After creating a facial landmark image by marking 68 facial landmark points as white dots on a black image, the facial landmark feature network and GAP were applied to obtain a facial landmark feature. The details of the facial landmark feature module are described later in Section 4.3. The resulting 48 facial image features and 48 facial landmark features were concatenated for each frame and then passed through the temporal attention module to obtain a final feature.

In the temporal attention module, a high weight was assigned to frame features that were highly related to stress, while a low weight was assigned to frame features that were less related to stress. The details of the temporal attention module are described later in Section 4.4. When the final obtained feature passed through the fully connected layer, a stress prediction result was finally produced. We divided the stress state into neutral, low, and high stress. Therefore, the stress prediction result would be one of these three states. While the learning was in progress, the part that was actually learned is marked with a red box in Figure 2.

### 4.2. Spatial Attention Module

**Figure 3:**
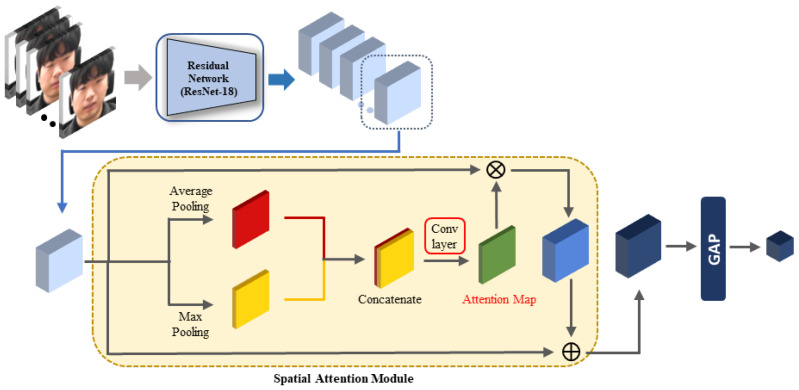
Structure of the spatial attention module. For efficient learning, the multiplication result from the original feature maps and the spatial attention map is added to the original feature maps. GAP: global average pooling.

Chen et al. [59] used a spatial attention module to pinpoint to the network the relevant parts of the feature map that should be viewed more closely. Since then, the spatial attention module’s structure has continued to develop. As the module proposed by Woo et al. [47] demonstrated both light and high performance, we used it to obtain the spatial attention weight. The spatial attention module’s overall structure is depicted in Figure 3, and the details are described below.

First, the feature maps were extracted by inputting the facial image into ResNet-18. This network is light and has high performance, so it is widely used in various recognition fields. We did not use a pretrained network; only the structure of ResNet-18 was used and trained from the beginning after initializing the weights. After obtaining the feature maps, average pooling and max pooling were performed on the channel axis. The two results were concatenated along the channel axis. Chen et al. [59] demonstrated that performing the pooling operation on the channel axis emphasizes locational importance. The average pooling operation used by Zhou et al. [60] is frequently used because it is effective for aggregating information.

Furthermore, Woo et al. [47] found that the max pooling operation reveals important information that differs from that revealed by the average pooling operation. Therefore, if the results obtained by performing both the average pooling and max pooling operations on the feature maps are concatenated and a convolutional operation is performed, it is possible to obtain an attention map that highlights stress-relevant regions by considering multiple perspectives. In our design, the sigmoid function was used to obtain the final spatial attention map. By multiplying the obtained spatial attention map by the original feature maps, feature maps with applied spatial attention can be obtained.

In the next step, the final feature maps were obtained by adding attention-applied feature maps to the original feature maps. This addition to the previous layer’s result is called identity mapping. This structure reduces the amount of information that the layer must learn so that learning can be performed more effectively [57]. Finally, the facial image feature was obtained by applying GAP to the final feature maps. While the learning was in progress, the part that was actually learned is marked with a red box in Figure 3. The facial image feature was obtained using the following equation:

(1) $$\begin{array}{c} M_{sa}=\sigma \left(conv^{7\times 7}\left(\left[AvgPool\left(F\right);MaxPool\left(F\right)\right]\right)\right)), \end{array}$$

(2) $$\begin{array}{c} f_{facial\;image}=\;GAP\left(F+M_{sa}\circ F\right), \end{array}$$

where σ is the sigmoid function, $conv^{7\times 7}$ denotes the convolutional operation with a 7×7 filter, *F* denotes the feature maps extracted from ResNet-18, the symbol; denotes the concatenation operation, GAP indicates the GAP operation, and ∘ is the product of the attention weight and feature value for each position in the feature map. The residual network’s structure and spatial attention module are depicted in Table 2. As can be seen from Table 2, the size of the feature space of the facial image feature was 4×4×512. This module was automatically trained through an end-to-end learning process. The importance of the spatial attention module is evaluated in Section 5.3.2.

### 4.3. Facial Landmark Feature Module

**Figure 4:**
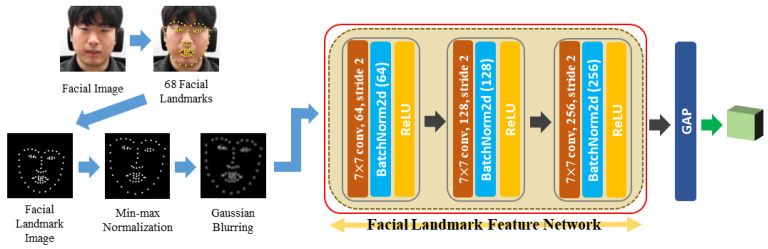
Facial landmark feature extraction process. A simple network with three convolutional layers is used to extract the facial landmark feature. GAP: global average pooling.

Giannakakis et al. [32] indicated that peoples’ eye, mouth, and head movements during stressful situations differ from those during nonstressful situations. To accurately capture these movements, we designed a network that receives facial landmark points representing the eye, mouth, and head positions as the input. The feature extracted from this network is used along with the facial image feature to complement its discriminating power. The process of extracting the facial landmark feature is depicted in Figure 4, and the details are described below.

A facial image was first input into the facial landmark detector to extract the facial landmark feature, where the detector was an ensemble of the regression tree algorithm [61]. Passing through the facial landmark detector, 68 facial landmarks were obtained and displayed as white dots on a black image to create a facial landmark image. The facial landmark image was used because it better captures the movement of the facial landmarks when input into the CNN, which uses spatial information, rather than simply entering the facial landmark coordinate values into the fully connected neural network. In the method proposed by Wu et al. [41], the facial landmark image was used to utilize the facial location, and it was shown that fine movements could be captured well. Therefore, we also tried to capture the minute movements of the face by proposing a method to utilize a facial landmark image by paying attention to this aspect.

Furthermore, two preprocessing steps were performed on the facial landmark image; one is min–max normalization, and the other is Gaussian blurring. Min–max normalization was used because the position of the area where the human face is detected in each frame of the video jitters slightly, so the face is stationary, but appears to be moving. If the location of the face area moves slightly, the location of the facial landmark detected in the facial area also moves slightly. Consequently, the head is stationary, but it may appear to move, which may adversely affect stress recognition. By performing min–max normalization, this phenomenon can be prevented because the positions of the facial landmarks are evenly aligned in all frames. In the face detection stage, we roughly aligned the positions of the eyes, nose, and mouth through alignment, but these positions were not always precisely fixed. Therefore, min–max normalization was additionally applied to reduce this phenomenon as much as possible.

After min–max normalization, Gaussian blurring was performed because jittering also occurred in the facial landmark detector result, and the effects that arise from these phenomena can be reduced when blurring is performed by spreading the data around a point rather than merely displaying that point. After performing these two preprocessing steps, the image was passed through the CNN. The structure of this network comprises three convolutional layers. The content of the facial landmark image is simple. Useful information can be extracted even by a simple network, so we chose a simple network to avoid unnecessary complexity. Finally, the facial landmark feature was obtained by performing a GAP operation on the feature maps that passed through the CNN. While the learning was in progress, the part that was actually learned is marked with a red box in Figure 4. The facial landmark feature module network structure is depicted in Table 3. As can be seen from Table 3, the size of the feature space of the facial landmark feature was 9×9×256.

### 4.4. Temporal Attention Module

**Figure 5:**
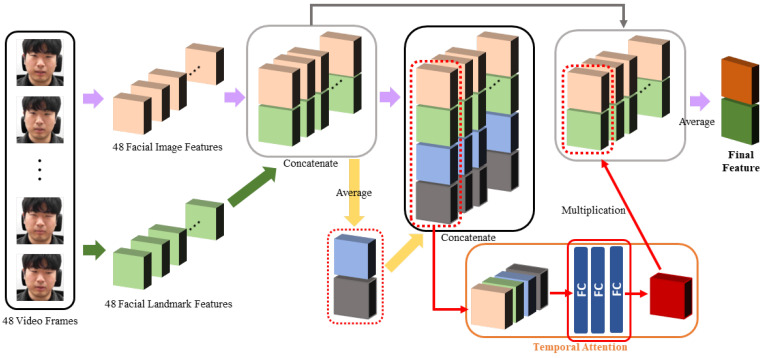
Structure of the temporal attention module. The attention weight increases when the frame is highly related to stress, considering the average feature representing 48 frames and the feature of a specific frame. FC: fully connected layer.

Meng et al. [49] used a temporal attention module to observe the information in all frames to determine on which frame to focus. As the structure is simple and demonstrated high performance in facial expression recognition, we modified this module and used it to obtain the temporal attention weight. The temporal attention module’s overall structure is depicted in Figure 5, and the details are described below.

First, 48 video frames passed through the ResNet-18 network and spatial attention module, and 48 facial image features were extracted. Then, these frames passed through the facial landmark detector and facial landmark feature network, and 48 facial landmark features were extracted. When the 48 extracted facial image features and 48 extracted facial landmark features entered the temporal attention module, they were first concatenated frame-by-frame to create 48 concatenated features. Thus, frames highly related to stress were found by considering the facial image features, as well as the facial landmark features.

The 48 concatenated features were averaged to obtain the average feature, and the average feature was concatenated into 48 concatenated features to generate 48 final concatenated features. The average feature can be regarded as containing all information for all frames. When the temporal attention weight is calculated using these final concatenated features, it becomes possible to obtain each frame’s temporal attention weight by comprehensively viewing the information of the entire frame, as well as the information of individual frames. Therefore, each final concatenated feature was passed through three fully connected layers to obtain each frame’s temporal attention weight. It is possible to attach the 49th slice and calculate the weight at once, but the weight of the target individual feature and the total feature decreases, so the desired weight value cannot be obtained. Therefore, we did not proceed in this manner.

When the obtained temporal attention weight for each frame is multiplied by the concatenated feature from the corresponding frame’s facial image feature and facial landmark feature, the concatenated feature reflects the importance of the corresponding frame. Accordingly, after obtaining the concatenated features that reflect the importance of all 48 frames, the final feature was obtained by applying the average operation. By applying a fully connected layer to this feature, the stress recognition result was output. While the learning was in progress, the part that was actually learned is marked with a red box in Figure 5. The final feature was obtained using the following equations:

(3) $$\begin{array}{c} f_{concat}^{i}=\left[f_{facial\;image}^{i};f_{facial\;landmark}^{i}\right], \end{array}$$

(4) $$\begin{array}{cc} f_{total\;concat}^{i} & =[f_{concat}^{i};Avg\left(f_{\mathrm{concat}}\right)], \end{array}$$

(5) $$\begin{array}{c} W_{ta}^{i}=fc^{1}\left(fc^{1536}\left(fc^{1536}\left(f_{total\;concat}^{i}\right)\right)\right), \end{array}$$

(6) $$\begin{array}{c} f_{final}=Avg\left(W_{\mathrm{ta}}\cdot f_{\mathrm{concat}}\right), \end{array}$$

where $f^{i}$ is the feature of the *i*th frame, Avg denotes the averaging operation on the time axis, $W^{i}$ is the weight of the *i*th frame, $fc^{n}$ represents a fully connected layer with *n* output nodes, and the symbol · denotes the multiplication operation for each frame. The bold notation indicates a vector of features or weights for all frames. The network structure of the temporal attention module is depicted in Table 4.

### 4.5. Loss Function

We trained and tested the proposed method using the constructed database. Given the database’s characteristics, the choice of the loss function influenced the training result considerably. For the constructed database, the difference in facial changes observed by the same person in different stress states is minute, so the difference between classes within the same subject’s data is not large.

In contrast, even in the same stress state, each person has a unique face, and a difference in the pattern of facial changes occurs. Accordingly, the difference between subjects within data from the same class is large. Therefore, if the distance between features for data from different classes within the data for the same subject is increased and the distance between features for data from different subjects within the data for the same class is decreased, it is possible to prevent ineffective learning caused by database characteristics.

Previous studies have proposed several loss functions to prevent this phenomenon, such as the widely used contrastive loss [62] and triplet loss [63] functions. For contrastive loss, only one positive data point and one negative data point are used in the loss function, but this may result in less efficiency than using both. For triplet loss, one formula handles both, reducing the distance between data for the same class and increasing the distance between data for different classes. However, this approach can reduce the learning ability when compared with methods that handle these tasks separately and then combine the results. Therefore, considering this information, we propose a new loss function by combining the two loss functions.

The first component of the proposed loss function reduces the Euclidean distance between the features extracted from the anchor data and the positive data to zero. The second component changes the Euclidean distance between the features extracted from the anchor data and the negative data to a value called the margin. The final loss function was completed by adding three cross-entropy losses to the proposed loss function. The three cross-entropy losses were obtained from the prediction scores of the anchor, positive, and negative data and the ground truth for each data point. The final loss function was obtained using the following equations:

(7) $$\begin{array}{c} L_{CE}=-\sum_{c=1}^{C}t_{c}\log\left(s_{c}\right), \end{array}$$

where *C* is the number of classes, $t_{c}$ indicates the ground truth of class *c*, and $s_{c}$ is the prediction score of class *c*.

(8) $$\begin{array}{c} L_{MSE}\left(f_{1},f_{2}\right)=\frac{1}{N}\sum_{i=1}^{N}{\left(f_{1}^{i}-f_{2}^{i}\right)}^{2}, \end{array}$$

(9) $$\begin{array}{cc} L_{final} & =L_{CE-anchor}+L_{CE-pos}+L_{CE-neg}\\ \; & +L_{MSE}\left(f_{anchor},f_{pos}\right)\\ \; & +\max(0,m-L_{MSE}\left(f_{anchor},f_{neg}\right)), \end{array}$$

where *N* denotes the feature dimension, $f^{i}$ is the *i*th element of the feature, and *m* represents the margin. Furthermore, $t_{c}$ is one when the ground truth of a data point is class *c* and zero for the rest, and $L_{CE-x}$ is the cross-entropy loss of *x* data.

Positive and negative data input into the final loss function were selected considering the characteristics of the constructed database. The positive data were selected to have the same class as the anchor data, with the selected subject being different from the anchor data. The negative data were selected to be a different class from the anchor data, with the selected subject being the same as the anchor data. The proposed method was learned end-to-end using this newly proposed loss function.

## 5. Experimental Results

This section explains the experiment we conducted. First, the experimental setting and dataset are described. Second, the results of the ablation study experiment performed to design the proposed method are presented. Finally, the results of the performance comparison experiment between the proposed method and other methods are explained and analyzed.

### 5.1. Experimental Setting

PyTorch, a deep-learning library, was used to implement the proposed method. We divided the training set and the testing set using a five-fold cross-validation method to evaluate the performance. When training, the parameters were set as follows. First, in the final loss function (9), the margin was set to 2, and for the optimizer, a stochastic gradient descent optimizer was used. The momentum was set to 0.9, and the weight decay was set to 0.0001. The training epoch was set to 45, and the initial value for the learning rate was set to 0.001 and decreased by 0.1 every 15 epochs. The batch size was set to maximize the GPU memory and set to 6 in the proposed method. We divided the data into a training set, a validation set, and a testing set in a ratio of 3:1:1, and the best hyperparameter set was determined by conducting experiments with various hyperparameter combinations for the validation set. During the division of the data, it was ensured that a subject’s data belonged to only one set, since if the same subject’s image were to be included in both the training and test sets, the subject’s appearance could be learned and the performance could hence be abnormally high.

In the experiments, the performance comparison between the methods used accuracy values obtained by dividing the number of correctly predicted clips in the testing set by the number of all clips in the testing set. As we used the five-fold cross-validation method, we used the average of five accuracy values from five testing sets.

### 5.2. Dataset

We used the Yonsei Stress Image Database previously described in Section 3 to evaluate the stress recognition performance. A total of 42,023 clips were created by dividing 2,020,556 images of 50 subjects into 48 consecutive frames, and the clips were used as the input for training and testing. The reason for defining a clip as 48 consecutive frames, i.e., two seconds in length, is as follows. In university labs, GPUs with 11GB of memory are often used. When learning the proposed method using this GPU, if 48 frames are input to the GPU, the maximum batch size is 6. If the batch size is too small, the performance deteriorates, so we could use up to 48 frames at once. Additionally, we conducted an ablation study (described in Section 5.3.4) to investigate the variation of the performance with the clip length. To match the experimental conditions as much as possible, 48 frames were randomly selected and used for clips longer than 2 s. In the experimental results, the 2 s clip showed the highest performance, so we used the 2 s clip as a training and test unit.

**Figure 6:**
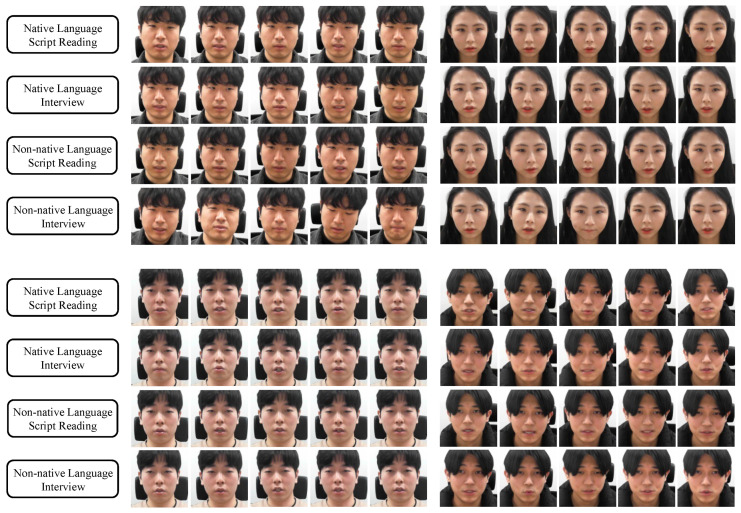
Samples of cropped facial images from the constructed database.

The facial images were cropped from the original images and input into the network. Examples of the facial images are depicted in Figure 6. For four randomly selected subjects, the various facial expressions displayed by them are presented for each situation.

### 5.3. Ablation Study

In this subsection, we describe the settings and results of the experiments conducted to select the structure of the proposed method. We also present the results of the experiments and examine the effect of the clip settings.

#### 5.3.1. Loss Function

First, an experiment was conducted to determine the loss function that most effectively improved the learning. The proposed loss function was designed with reference to the contrastive loss [62] and triplet loss [63] to ensure effective learning considering the characteristics of these databases. The performance was compared with these functions to determine whether the proposed loss function was effective. The results are listed in Table 5.

As depicted in the experimental results, the best performance was achieved when the cross-entropy loss and proposed loss were used together. When learning using the proposed loss function, the distance between the data from the same class was reduced, and the distance between the data from different classes was increased when compared with using other loss functions.

#### 5.3.2. Attention Module

With several types of attention modules available, we experimented to determine the best combination by fusing several attention modules. The attention modules used in the experiment are common: the spatial attention module, channel attention module, and temporal attention module. The spatial and channel attention modules were proposed by Woo et al. [47], and the temporal attention module was a modified version of that proposed by Meng et al. [49]. Table 6 presents the experimental results for various combinations of attention modules.

The experimental results demonstrated that the highest performance occurred when the spatial attention and temporal attention modules were both used. Accordingly, finding a channel with a high correlation to stress on the feature maps did not significantly affect the performance, whereas finding a location and frame with a high correlation to stress significantly affected the performance.

#### 5.3.3. Facial Landmark Feature Module

Furthermore, 68 facial landmarks were imaged and entered into the network to extract facial landmark features, and an experiment was conducted to determine the best method for processing and inputting these facial landmark images. As the results of the face detector and facial landmark detector illustrated a jittering pattern, we examined the extent to which the stress recognition performance was affected when this phenomenon was prevented by applying min–max normalization and Gaussian blurring to the facial landmark images. The experimental results are listed in Table 7.

The experimental results demonstrated that the performance decreased when only the landmark image was used or only min–max normalization was applied. However, when min–max normalization and Gaussian blurring were both applied to the landmark images, the performance increased. Thus, when both min–max normalization and Gaussian blurring were used, the jittering phenomenon was prevented.

#### 5.3.4. Clip Length and Number of Frames

Finally, we analyzed the impact of the proposed method on the performance by varying the clip length and number of frames. First, we experimented by changing the clip length, which is a unit used in training and testing, to 1 s, 2 s, 5 s, 10 s, and 30 s; the results are listed in Table 8. To match the experimental conditions as much as possible, we used 24 frames for 1 s, and 48 frames were used in the remaining experiments.

The experimental results demonstrated that the best performance occurred when the clip length was 2 s. It was possible to identify the cues that indicated stress in 2 s clips, and the temporal change was learned well using 48 consecutive frames. In contrast, we randomly selected 48 frames for clips longer than 2 s and used them for training and testing; hence, the discontinuity between frames could have an adverse effect on learning the temporal change. Next, we experimented by changing the number of frames constituting one clip to 8, 16, 32, 48, and 64, and the results were the same as in Table 9. To match the experimental conditions as much as possible, we used 2.7 s clips for 64 frames, while the other experiments used 2 s clips.

The experimental results demonstrated that the highest performance was achieved when 48 frames were used. This setting exhibited the highest performance when all 48 frames of the 2 s clips were used because it is necessary to find the overall spatial and temporal facial changes when recognizing stress. In contrast, when the clip length exceeded 2 s, recognition was hampered by the increased amount of unnecessary information, as in the above experiment.

### 5.4. Comparison with Other Methods

We evaluated the stress recognition performance of the proposed method, as well as various other methods. We compared the proposed method with widely used deep-learning networks that have demonstrated high performance [46,47,57,64,65,66,67]. The HOG–SVM method, which combines the widely used handcrafted features, HOG [68], and the classical classifier SVM [69], was used for comparison. In addition, current deep-learning-based recognition methods [41,42,43,45] using spatial–temporal facial information were also used for performance comparison. These methods were used because an emotion recognition network could be considered similar to a stress recognition network.

The experimental results of the proposed method and other methods are listed in Table 10, along with each method’s feature dimension. In general, a higher feature dimension indicates a higher discriminating power, but because the computational complexity increases, lower feature dimensions that exhibit high performance are preferable.

As depicted in the experimental results, the proposed method had the highest accuracy, 66.8409%, even though features with a relatively low dimensional number of 768 were used. Even when the facial landmark feature was not used, it exhibited an accuracy of 65.3396% with a small 512-dimensional feature. SE-ResNet-18 had the highest performance, at 65.7013%, among the widely used deep-learning networks. This network uses attention modules, which seems to have a positive effect on the stress recognition performance.

By contrast, VGG-16 and ResNet-50 exhibited low performance despite using a relatively high number of feature dimensions, i.e., 2048. This result demonstrates that these methods have a network structure that is unsuitable for stress recognition. The HOG–SVM method used a relatively high number of feature dimensions, i.e., 1764, but exhibited the lowest performance, i.e., 50.9153%. Thus, it was demonstrated that the discriminating power of the handcrafted features was lower than that of the deep-learning networks.

Examining the results of methods using spatial–temporal facial information, the method using the 2D-CNN, LSTM, and facial landmarks demonstrated low performance, i.e., 58.3432%. This result indicates that the facial landmark information was not utilized satisfactorily because the coordinates of the facial landmarks were simply input into the network. Furthermore, the method using the 3D-CNN with hyperparameter optimization exhibited high performance at 65.9372%. Thus, even a simple network can exhibit high performance through appropriate hyperparameter optimization.

We also compared the performance with the method using a physiological signal database [13]. It can be seen that the performance of that method was higher than ours at 74.1%. However, unlike our method, which classified three stress states, this method classified two stress states. In addition, since this method uses physiological signal data, a direct comparison with our method is not possible. Therefore, our approach, which showed the highest performance when there were three stress states, was quite competitive as it offered finer distinctions. Furthermore, as mentioned before, our method does not require biosensors and has the advantage of being able to be used for more diverse applications using images.

Furthermore, we compared the performance with the previous video-based stress-recognition method [30]. The performance of the method was high at 64.6481%, but the performance was lower than that of our proposed method. Therefore, the experimental results in Table 10 show that the performance of the proposed method was higher than that of the other methods. These results indicate that the proposed method is superior to other methods in detecting the overall spatial and temporal changes of the face.

We present the sensitivity and specificity rates along with classification accuracy in Table 10. It can be confirmed that the proposed method showed the best performance in both sensitivity and specificity, as well as accuracy.

**Figure 7:**
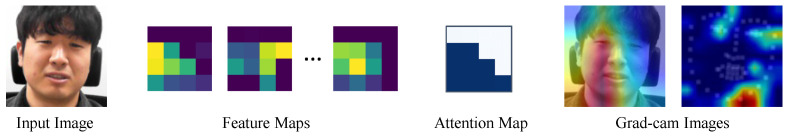
Feature maps, attention map, and Grad-cam images output from an example facial image.

We also output the feature maps and attention map obtained from the spatial attention module, and the results are shown in Figure 7. In the case of the attention map, it can be seen that a higher weight was assigned to the lower part of the face. However, in the case of the feature map, it can be seen that it is difficult to identify which features have been learned because the resolution was as low as 4 × 4. Therefore, we drew a picture of the Grad-cam [70], which shows which part of the image was mainly viewed and determined the prediction. We drew the Grad-cam results for the facial image, as well as the facial landmark image, and the results are shown in Figure 7. As can be seen from Figure 7, the network predicted the stress level primarily by considering the areas around the eyes and mouth.

**Figure 8:**
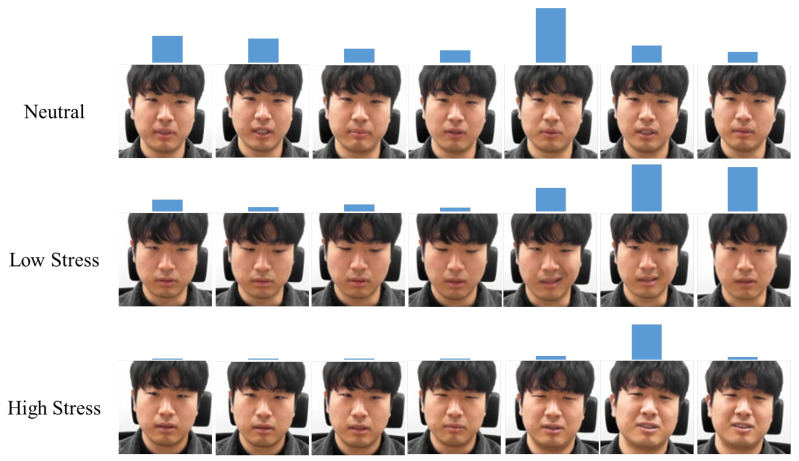
Visualization of the temporal attention weight in three stress states. The higher the height of the bar on the image, the greater the weight is.

In addition, temporal attention weights were visualized to check whether temporal attention was well applied, and the result is as shown in Figure 8. In the neutral state, the change rate of the weight was not large; however, in the stressed state, the change rate was large. It can be seen that the weight was higher for images in which the change in facial expression was large. This showed that the temporal attention module was working properly.

**Figure 9:**
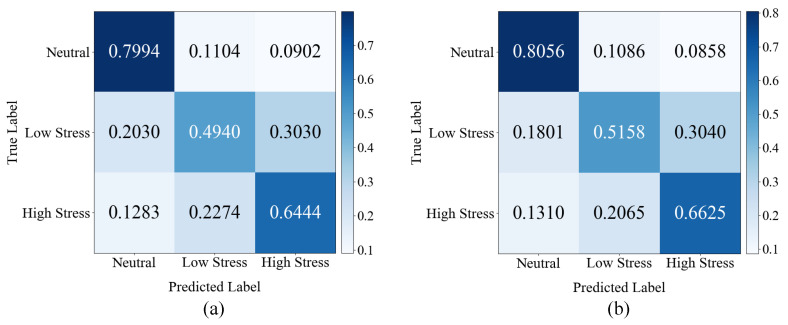
Confusion matrix of the proposed method (**a**) without and (**b**) with the facial landmark feature.

The classification accuracy for each of the proposed method’s classes is listed in Table 11. When the facial landmark feature was used, the proposed method demonstrated higher performance for all three classes than when it was not used. This result implies that the facial landmark feature effectively complements the facial image feature. However, even if the facial landmark feature is used in the proposed method, its classification of the neutral state was superior to its classification of the stress states. Thus, it is challenging to find overall spatial and temporal facial changes that appear when people are under stress. Especially under low stress, the changes are smaller, so they are more difficult to pinpoint. We also output the confusion matrix of the proposed method without and with the facial landmark feature, and the results are shown in Figure 9. Figure 9 shows that the overall performance improved when the facial landmark feature was used compared with not using it.

**Figure 10:**
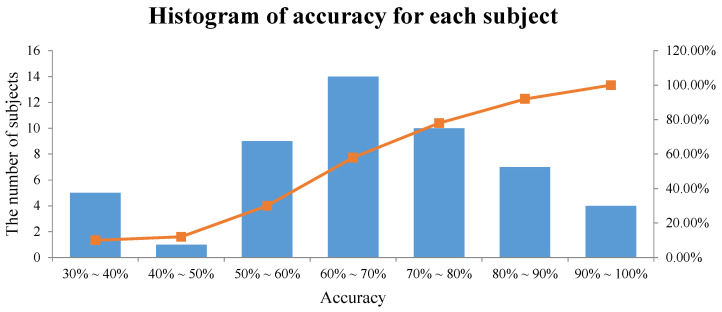
Histogram of the accuracy for each subject in the proposed method.

We plotted a histogram of the accuracy of each subject in the proposed method, as shown in Figure 10. The histogram shows how the average performance of the three classes is distributed for all subjects. More specifically, five subjects with an accuracy of 30%∼40% means that the number of subjects with an average performance of three classes between 30% and 40% is five. The interval with the largest number of subjects was between 60% and 70%, and the average performance of the three classes in our method from Table 11 also involved this interval.

To evaluate the performance of the video unit, we performed classification by dividing all 5 min and 10 min videos into 2 s clips. For each subject, there were two 5 min videos for the neutral class and one 10 min video for the low- and high-stress classes. If the ratio of correctly classified clips was greater than the threshold, the video was counted as correctly classified and the accuracy was measured. The video unit performance of the proposed method is shown in Table 12, and it is possible to grasp the trend of the performance change according to the threshold change. Since the accuracy was calculated using the results of Table 10 learned by the cross-validation method, the cross-validation method was also applied to these results. If the threshold was set to 50%, the video unit performance was better than the 2 s clip unit performance. For the three classes, the threshold value of 50% can be seen as a reasonable value.

## 6. Conclusions

In this paper, a stress-recognition method using spatial–temporal facial information was proposed using deep learning. To use deep learning technology, we built and released a large image database for stress recognition. In the proposed method, we used a spatial attention module that assigns a high weight to the stress-related regions of the facial image. Using a temporal attention module that assigns a high weight to frames that are highly related to stress from among several frames in the video, we improved the feature’s discriminating power. Furthermore, using features extracted from the facial landmark information, we supplemented the discriminating power of the feature extracted from the facial image.

We designed the loss function so that the network learning proceeds effectively, considering the characteristics of the constructed database. We evaluated the proposed method on our constructed database, and it exhibited higher performance than existing deep-learning-based recognition methods. However, our approach has a limitation in that it would find it difficult to recognize stress in people who do not display much change in their facial expressions. In the future, to mitigate this limitation, a study on stress recognition based on multimodal data will be conducted using voice data, which is closely related to stress, along with the images. In addition, research in more difficult environments such as occlusion on the face will be conducted as future work.

## Figures and Tables

**Table 1:** Number of images acquired at each stage of the database construction.

Designed State	Experimental Stage	Total Images
Neutral	Native Language Script Reading	366,121
	Non-native Language Script Reading	368,991
Low Stress	Native Language Interview	656,624
High Stress	Non-native Language Interview	628,820

**Table 2:** Network structure of the residual network and spatial attention module.

	Unit	Layer	Filter/Stride	Output Size
Input	0			112 × 112 × 3
Residual Network	1	Conv-BN-ReLU	7 × 7, 64/2	56 × 56 × 64
		Max Pooling	3 × 3/2	28 × 28 × 64
	2	Conv-BN-ReLU	3 × 3, 64/1	28 × 28 × 64
		Conv-BN	3 × 3, 64/1	28 × 28 × 64
	3	Conv-BN	1 × 1, 128/2	14 × 14 × 128
		Conv-BN-ReLU	3 × 3, 128/1	14 × 14 × 128
		Conv-BN	3 × 3, 128/1	14 × 14 × 128
	4	Conv-BN	1 × 1, 256/2	7 × 7 × 256
		Conv-BN-ReLU	3 × 3, 256/1	7 × 7 × 256
		Conv-BN	3 × 3, 256/1	7 × 7 × 256
	5	Conv-BN	1 × 1, 512/2	4 × 4 × 512
		Conv-BN-ReLU	3 × 3, 512/1	4 × 4 × 512
		Conv-BN	3 × 3, 512/1	4 × 4 × 512
Spatial Attention Module	6	AvgPool		4 × 4 × 1
		MaxPool		4 × 4 × 1
		AvgPool + MaxPool		4 × 4 × 2
		Conv-Sigmoid	7 × 7, 1/1	4 × 4 × 1
	7	Product (5 ∘ 6)		4 × 4 × 512
Output	8	GlobalAvgPool		512

BN: batch normalization. In Unit 7, the outputs of Units 5 and 6 are multiplied for each position in the feature maps.

**Table 3:** Network structure of the facial landmark feature module.

	Unit	Layer	Filter/Stride	Output Size
Input	0			112 × 112 × 1
Facial Landmark Feature Network	1	Conv-BN-ReLU Conv-BN-ReLU Conv-BN-ReLU	7 × 7, 64/2 7 × 7, 128/2 7 × 7, 256/2	53 × 53 × 64 24 × 24 × 12 89 × 9 × 256
Output	2	GlobalAvgPool		256

BN: batch normalization. The stride is 2, but the feature map size is reduced by more than 0.5-times because padding is not performed during convolution.

**Table 4:** Network structure of the temporal attention module.

	Unit	Layer	Output Size
Input	0	Facial Image Feature	512 × 48 (frames)
	1	Facial Landmark Feature	256 × 48 (frames)
Temporal Attention Module	2	Concatenate (0 + 1)	768 × 48 (frames)
	3	Average (48 frames)	768
	4	Concatenate (2 + 3)	1536 × 48 (frames)
	5	Fully Connected	1536 × 48 (frames)
		Fully Connected	1536 × 48 (frames)
		Fully Connected	1 × 48 (frames)
	6	Multiplication (2 · 5)	768 × 48 (frames)
	7	Average (48 frames)	768
Output	8	Fully Connected	3 or 4

In Unit 4, the outputs of Units 2 and 3 are concatenated for each frame. In Unit 6, the outputs of Units 2 and 5 are multiplied for each frame.

**Table 5:** Comparison of different loss functions.

Method	Accuracy (%)
ResNet-18 + Cross-Entropy Loss	60.0895
ResNet-18 + Cross-Entropy Loss + Contrastive Loss	63.1357
ResNet-18 + Cross-Entropy Loss + Triplet Loss	62.8771
ResNet-18 + Cross-Entropy Loss + Proposed Loss	64.1865

**Table 6:** Comparison of various combinations of attention modules.

Method	Accuracy (%)
ResNet-18	64.1865
ResNet-18 + Spatial Att	64.7608
ResNet-18 + Channel Att	65.1097
ResNet-18 + Temporal Att	65.2569
ResNet-18 + Channel Att + Temporal Att	64.3173
ResNet-18 + Spatial Att + Temporal Att	65.3396
ResNet-18 + Spatial Att + Channel Att	64.4165
ResNet-18 + Spatial Att + Channel Att + Temporal Att	64.8969

**Table 7:** Comparison of facial landmark feature extraction methods.

Method	Accuracy (%)
ResNet-18 + Att	65.3396
ResNet-18 + Att + Landmark Image	63.0085
ResNet-18 + Att + Landmark Image + Norm	64.0012
ResNet-18 + Att + Landmark Image + Blur	66.1854
ResNet-18 + Att + Landmark Image + Norm + Blur	66.8409

Att: spatial and temporal attention modules, Norm: min–max normalization, Blur: Gaussian blurring.

**Table 8:** Effect of clip length on the performance.

Method	Clip Length (s)	Accuracy (%)
	1	65.9470
	2	66.8409
Ours	5	65.6555
	10	65.8282
	30	65.6207

**Table 9:** Effect on the performance of the number of frames.

Method	Number of Frames	Accuracy (%)
	8	65.0138
	16	64.8687
Ours	32	66.1900
	48	66.8409
	64	64.4527

**Table 10:** Stress recognition accuracy, sensitivity, and specificity on the constructed database.

Method	Feature Dimension	Accuracy (%)	Sensitivity (%)	Specificity (%)
HOG-SVM [68,69]	1764	50.9153	50.4360	64.3488
VGG-16 [65]	2048	56.9125	56.4093	71.0178
CBAM-ResNet-18 [47]	512	58.8559	58.1435	72.4161
ResNet-50 [57]	2048	60.0093	59.4649	74.2789
ResNet-18 [57]	512	60.0895	59.4573	74.4877
Inception v3 [66]	2048	63.4185	62.8578	77.6015
AlexNet [64]	4096	64.1588	63.4871	78.3340
DenseNet-121 [67]	1024	64.9408	64.4349	78.2179
SE-ResNet-18 [46]	512	65.7013	65.1206	79.2945
2D-CNN + LSTM + Facial Landmark [42]	768	58.3432	57.7521	72.5148
3D-CNN + Facial Landmark Image [41]	4096	62.5361	62.1877	76.1710
2D-CNN + GRU + Multimodel [43]	512	65.8770	65.3907	79.3543
3D-CNN + Hyperparameter Optimize [45]	4096	65.9372	65.4369	79.7895
Zhang et al. [30]	47,104	64.6481	64.0199	78.3209
Ours (w/o Facial Landmark Feature)	512	65.3396	64.5928	78.9639
Ours	768	66.8409	66.1292	80.0959

**Table 11:** The proposed method’s classification accuracy for each stress state with and without the facial landmark feature.

Stress State	Accuracy (%)	
	Ours (w/o Facial Landmark Feature)	Ours
Neutral	79.9396	80.5567
Low Stress	49.4030	51.5811
High Stress	64.4358	66.2499

**Table 12:** Video-based stress recognition accuracy in the proposed method obtained by changing the threshold.

	Accuracy (%)		
Threshold	40%	50%	60%
Neutral	84.0000	79.0000	77.0000
Low Stress	58.0000	56.0000	52.0000
High Stress	79.5918	73.4694	67.3469
Total	73.8639	69.4898	65.4490

## Data Availability

The data presented in this study are openly available on IEEE DataPort at https://dx.doi.org/10.21227/17r7-db23 (accessed on 8 November 2021).

## Appendix A

**Table A1:** Summary of the database construction settings and database contents.

Item	Description
Number of Subjects	50
Age of Subjects	20–39 y
Gender Ratio of Subjects	1:1
Nationality of Subjects	Korea
Number of Experimental Stages	4
Number of Stress States	3
Number of Videos per Subject	4
Camera Used for Recording	Kinect v2
Image Resolution	1920 × 1080
Data Acquisition Rate	24 frames/s
Total Number of Images	2,020,556
Total Length of Recorded Videos	1403 min
Illumination	Keep the lights constantly bright
Background	Only clean, white walls
Head Orientation	Almost straight ahead
Occlusion	Hair or accessories do not cover the face (excluding glasses)
